# A Conceptual Model for Understanding the Division and Transfer of Diabetes Care Responsibilities Between Parents and Children with Type 1 Diabetes

**DOI:** 10.3390/healthcare13101143

**Published:** 2025-05-14

**Authors:** Jori Aalders, Frans Pouwer, Esther Hartman, Giesje Nefs

**Affiliations:** 1 Center of Research on Psychological Disorders and Somatic Diseases [CoRPS], Department of Medical and Clinical Psychology, Tilburg University, 5037 AB Tilburg, The Netherlands; e.e.hartman@tilburguniversity.edu (E.H.); giesje.nefs@radboudumc.nl (G.N.); 2 Department of Psychology, University of Southern Denmark, 5230 Odense, Denmark; 3 Steno Diabetes Center Odense, 5260 Odense, Denmark; 4 Department of Health and Caring Sciences, Western Norway University of Applied Sciences, 5063 Bergen, Norway; 5 School of Psychology, Deakin University, Geelong, VIC 3216, Australia; 6 Diabeter, Center for Focused Diabetes Care and Research, 3013 TA Rotterdam, The Netherlands; 7 Radboud University Medical Center, Department of Medical Psychology, 6500 HB Nijmegen, The Netherlands; 8 Diabeter Centrum Amsterdam, 1066 EC Amsterdam, The Netherlands

**Keywords:** diabetes, paediatric, self-care, responsibility, transfer, review

## Abstract

**Background/Objectives**: For families with a child with type 1 diabetes, it is often complex and challenging to decide how responsibilities for diabetes care should be divided between parents and children, and how and when these responsibilities should be transferred from parent to child. A smooth transfer of responsibilities is assumed to be key for optimal diabetes outcomes and a successful shift from paediatric to adult health care. However, a theoretical framework to conceptualise the division and transfer of diabetes care responsibilities that brings together the scattered literature regarding these topics is still lacking. **Methods**: This narrative review synthesises insights from (a) prior quantitative and qualitative studies in the context of paediatric diabetes care, (b) prior reviews regarding the transfer of treatment responsibilities for families of children with a chronic condition, and (c) existing theoretical models in paediatrics, child development and parenting. **Results**: The division of responsibilities appears to be affected by a complex interaction between child, parent and context characteristics. These factors seem to change the division of diabetes care responsibilities by affecting (1) child/parental readiness to assume responsibility, (2) the alignment between the child’s and the parent’s readiness and (3) context support and demands. The “success” of the division and transfer of diabetes care responsibilities can be defined by biomedical, emotional, behavioural and parent–child interaction outcomes. **Conclusions**: The presented conceptual framework can guide research and clinical practice in studying and evaluating the division and transfer of diabetes care responsibilities.

## 1. Introduction

Achieving independence is an important developmental task that starts in childhood and continues over adolescence and young adulthood, asking multiple adaptations from both children and parents [1,2]. This process is even more complex in the context of paediatric type 1 diabetes, where families have the additional task of transferring the responsibility for diabetes care against the backdrop of maintaining physical and mental health [3]. Clinical guidelines only provide global recommendations that merely describe what can be expected in terms of child knowledge and practical skills for certain age groups [4,5,6,7,8,9], but do not elucidate how responsibilities can be best divided or renegotiated between parents and children. Moreover, in prior studies, the division and transfer of diabetes care responsibilities appeared to be dependent on factors other than just the child’s age [10]. However, a theoretical framework to conceptualise the division and transfer of diabetes care responsibilities and bring together the scattered literature regarding this topic is still lacking.

Therefore, the aim of this narrative review is to develop a conceptual model of the division and transfer of care responsibilities in paediatric type 1 diabetes for use in research and clinical practice. To this end, we will discuss the following sections respectively: (1) definitions relevant to the concepts of “division” and “transfer” of diabetes care responsibilities; (2) an overview of predictors/correlates of the division and transfer of diabetes care responsibilities drawn from earlier quantitative and qualitative work as well as information from existing theoretical models in paediatrics and the broader child development and parenting literature to aid in understanding; (3) how to determine the most optimal division and transfer of care responsibilities; and (4) integration of these insights into an overarching conceptual model.

## 2. Materials and Methods

This narrative review is based on (a) prior quantitative and qualitative studies in the context of paediatric diabetes care, (b) prior reviews regarding the transfer of treatment responsibilities in families of children with a chronic condition, and (c) existing theoretical models in paediatrics and the broader child development and parenting literature. To identify relevant prior quantitative and qualitative studies, literature searches were performed in PubMed and PsycINFO in 2024. The keywords and Mesh terms related to the following search terms were combined by using the Boolean operator “AND”: (1) type 1 diabetes, (2) children/adolescents, (3) parents, (4) responsibility/transfer. Empirical studies that were published before the 1st of January 1990 were excluded, as diabetes self-care has changed substantially in recent decades. Only peer-reviewed full-text studies written in English were considered. Studies were considered relevant if the study sample consisted of children with type 1 diabetes between the ages 6–18 years and/or their parents.

Reviews and theoretical models were selected based on face-value relevance to the division and transfer of diabetes care responsibilities (e.g., discussing ways to describe child readiness, parent readiness or the alignment between parent and child roles). Included references and citing articles were screened for studies that were relevant to ensure completeness.

## 3. Results

### 3.1. Definitions

Table 1 provides an overview of the concepts defined in this section.

#### 3.1.1. The Division of Diabetes Care Responsibilities

##### An Overview of Diabetes Self-Care

Self-care for type 1 diabetes is an intensive and complex round-the clock balancing effort, appealing to multiple skills across multiple activities [9]. Direct tasks include frequent monitoring of glucose levels, injecting insulin through an insulin-pen or insulin pump (while taking into account a range of key factors such as food intake, physical activity level, ambient temperature, illness-factors such as fever or vomiting, quality and duration of sleep and emotional stress levels), and recognising and self-treating hypo- or hyperglycaemia (low or high glucose levels, respectively) [19]. Additional indirect tasks include the management of medical, technological and food supplies needed for diabetes self-care, consulting diabetes health professionals, and communicating with others (such as school personnel and the parents of friends) about the condition and its management [19]. Next to the behavioural performance of self-care tasks, more recent studies have endorsed decision making as a separate skill dimension, for example with respect to the timing and amount of insulin or carbohydrates [20]. Another cognitive activity that is often overlooked or studied in isolation is recalling, which includes actually remembering to take required actions as well as prioritising diabetes care tasks over other activities when needed. This means that diabetes care consists of a collection of direct and indirect tasks across the domains of recalling, deciding and performing. These self-care tasks are part of the broader active, daily and flexible process of “self-management” to manage the condition in the context of one’s life in order to achieve and maintain both health and well-being [19].

##### Type 1 Diabetes Self-Care in the Paediatric Context

As the required skills for most diabetes care activities cut across several dimensions (i.e., motor, cognitive and emotional skills) that are still under development in childhood and adolescence, “self-care” (and “self-management”) in the paediatric context does not only involve the child who has the condition, but also family members, care-takers and significant adults in other contexts such as school teachers or sports coaches [17]. This translates to a *distribution* or *division* of diabetes care tasks between adults and children at a certain point in time, for example at the time of diagnosis [21].

##### Diabetes Care Responsibilities

Contrary to studies focusing on the extent to which treatment recommendations of the health care team are followed irrespective of actor (previously referred to as “adherence” or “compliance”) [22], the division of diabetes care tasks focuses on who actually executes diabetes self-care tasks. The division of diabetes care tasks is frequently studied in the broader context of *diabetes care responsibilities*. In general, *responsibility* has been defined as the duty or obligation of dealing with something [23], as well as “thoughtful compliance oriented towards achieving the objective of the norm or meeting one’s obligations to others rather than towards avoidance of blame or superficial conformity” [24]. What “should” transpire and what makes a person a responsible individual is largely dependent on the current sociocultural and historical context [25]. While terms like “compliance” and “should” fail to do justice to the complexity of living with a chronic condition [22,26], these more general definitions do highlight that it is important to verify what individuals consider to be “the norm” and “responsible”.

Parents are likely to define “being responsible” in self-care in adult terms (e.g., following treatment recommendations, taking into account long-term consequences of behaviour), while child behaviours that are in these parental terms considered as “not being responsible” could also originate from the child acting as a “responsible being” with respect to other life domains (e.g., not limiting one’s dreams of the future based on the need to manage a chronic illness) [17]. Therefore, it has been proposed to not restrict the definition of “being responsible” in paediatric health care to the extent to which treatment recommendations are followed, but to characterise a person who accepts responsibility as one who: (1) thinks rationally and prioritises others’ interests above his/her own interests, (2) holds a long-term view and is not solely oriented toward short-term outcomes, (3) defines core and side issues by discussion, (4) supervises how much effort is needed in a certain situation and (5) accepts the consequences of their actions [17,24].

##### A Note on “The Eye of the Beholder”

As perceptions about when responsibility is taken may differ between parents and children, it is unsurprising that within parent–child dyads, reports about the perceived division of diabetes care responsibilities are often inconsistent between parents and their children [27,28,29,30,31,32]. These conflicting views about the division can also arise if a child feels that he/she takes responsibility for a certain task, while parents perceive it is too early for the child to carry the responsibility. For example, when the child gives bolus without consulting a parent. It is also possible that perceptions differ from actual diabetes management. For example, parents may have decided to give the child permission and children agree that they themselves have responsibility for a certain task, yet parents continue to bear responsibility because the children neglect to assume this responsibility. With regard to assuming responsibilities, parental perceptions are limited to their child’s observable active behaviours. For parents, it might be difficult to get an overview of what children choose *not* to do in order to take responsibility for diabetes care (e.g., avoidance of physical exercise when blood glucose values are low) [17]. On the other hand, children might underestimate the involvement of parents in diabetes care, as they are not yet fully aware of all diabetes care tasks. For example, if the parent and child order diabetes supplies together but the parent additionally contacts the insurance company and diabetes team about supplies, children may perceive that the responsibility for managing supplies is shared while their parents perceive it as a parental responsibility.

#### 3.1.2. The Transfer of Diabetes Care Responsibilities

##### Goals of Transfer

Over time, diabetes care tasks and responsibilities are transferred from adults to children, mostly within the parent–child partnership. Therefore, this process can be viewed from both a child’s and a parent’s perspective, with different goals for both actors.

Child Perspective

In the broader literature about the management of chronic conditions, the end point for children in the transfer of responsibilities has been defined as “independent administrator” (i.e., initiating and implementing health care tasks generally out of the sight of or without the supervision of a parent) [33] and “being independent” or “autonomous” [11,20]. Within the literature, independence and autonomy have been differently conceptualised [34,35]. For example, autonomy has been described within developmental theories as taking distance from parents (separation) while taking responsibility without being dependent on parents (independence), but it also has been defined as self-government based on personal interest, values and goals [12,13,15,35]. Across theoretical perspectives on autonomy and independence, functional, attitudinal and emotional dimensions have been identified, relating to, respectively, the regulatory (strategy development), cognitive (decision making, goal definition) and affective (confidence in own choices and goals) aspects of behaviour [34].

Within paediatric diabetes care, Hanna and Decker (2010) defined the process of “assuming responsibility for self-care” as “a process specific to diabetes within the context of development that is gradual, daily, individualised and unique to the task, with the goal of ownership that involves autonomy in behaviours and decision making” [20]. In the context of type 1 diabetes, the term “ownership” implies that persons are “not only responsible for their type 1 diabetes, but that they also actively fulfilling that responsibility” [14]. Like autonomy and independence, ownership requires a combination of behavioural, emotional and cognitive efforts, in order to commit and strive to take care of one’s condition [14]. According to this definition of ownership, the endpoint of the transfer of diabetes care responsibilities as defined by Hanna and Decker (2010) (“full ownership that involves autonomy in behaviour and decision making”) is reached when “having/bearing” and “assuming/taking” responsibility for all diabetes care behaviours and decisions are united; children act autonomously and responsibly in diabetes care and they feel it is their own obligation to do it [20]. In the context of the emerging adulthood transition, Hanna (2012) similarly coined the term “primary diabetes care responsibility” as a developmental outcome [16].

In a different light, it has also been argued that during adolescence and emerging adulthood, the interdependent relationship between the child and parent(s) in diabetes care shifts to interdependent relationships with others, like friends or romantic partners, instead of the child becoming solely responsible for diabetes management [36].

The goal of transfer is ideally reached before children spend more time outside home (e.g., working, going to college, moving out), when parents are less available to provide instrumental support for diabetes care. Moreover, significant levels of autonomy and ownership have been considered as important indicators for a successful transfer from paediatric to adult health care [14,18]. However, some authors argue that throughout adolescence, shared responsibility is needed to optimise diabetes care outcomes [37], and the child gains primary diabetes responsibility during the early adulthood years [16].

Parent Perspective

In developmental psychology, parents are highlighted as the agents through which children develop responsibilities [38,39]. Until children develop the ability to assume responsibility, parents are considered to be responsible for the child’s physical health and emotional well-being. In a qualitative study examining responsibility for conducting at-home physiotherapeutic exercises in families with a child with cystic fibrosis, “non-involvement” was described as the final stage for parents in the transfer of responsibility [33]. At the same time, this may not imply that parents have completely withdrawn from the child’s health care [33]. Like other domains where the parent transfers responsibilities to the child (e.g., household, personal hygiene), the parent–child relation is expected to change into an adult-to-adult relation [33]. Next to friends and partners, parents can still provide emotional and instrumental support to their children on request, but they are no longer responsible for the overall quality and outcomes of diabetes care. The process is often termed “letting go” [10], although this term does not reflect the complexity of this parental task.

##### Course of Transfer

The process towards “full ownership” or “independence” for children and “letting go of responsibilities” for parents appears to occur gradually [20,33]. In a qualitative study among parents of children aged 9–14 years with type 1 diabetes [40], a general description of the course of the transfer was provided. Parents reported that direct tasks were usually transferred before indirect tasks. Furthermore, tasks in the domain of performing were in general the first transferred to the child, followed by tasks involving decision making and memorising. In this study, parents described the transfer process as a non-linear, dynamic, ongoing process with many challenges and one that is at times stressful. One of the parents used the metaphor of a yo-yo to describe the course of the process: children become more independent over time, yet parents repeatedly have to temporarily retake responsibilities.

In a qualitative study about the transfer of responsibilities for physiotherapy among families with a child with cystic fibrosis, separate child and parental roles through which children and parents move within the transfer process were identified [33]. These roles may also apply in diabetes care, although the content of self-care tasks differs and families can enter halfway through this process, as type 1 diabetes is often diagnosed in later childhood or adolescence [41,42].

Child Perspective

For children, five different roles with gradually increasing child responsibility were defined in the transfer process: (1) overwhelmed recipient, (2) partial recipient, (3) partial implementer, (4) non-initiating implementer and (5) independent administrator [33]. Children need to develop several physical, cognitive and emotional skills in order to be able to perform diabetes care tasks and to shift roles [9]:

- Fine and gross motor skills, for example to perform blood glucose measuring or to operate an insulin pump;
- The ability to recognise symptoms of hypo- and hyperglycaemia and to communicate how they feel to others;
- Cognitive skills, such as understanding numbers and numeric ordering (e.g., with respect to glucose monitoring results), advanced mathematical skills (e.g., to calculate the insulin dose for food), understanding of cause-and-effect relations (e.g., to determine the amount of insulin to administer), and abstract thinking (e.g., to anticipate, manage and prevent hyper- and hypoglycaemia);
- Emotional regulation skills to deal with diabetes care tasks in a responsible way. For example, to perform an unpleasant action like fingerpricks, to deal with uncomfortable reactions from others, and to cope with frustration and motivation problems arising from self-managing an unpredictable condition such as type 1 diabetes.

Parent Perspective

For parents, six different roles with gradually decreasing parental responsibility were defined: (1) completely directing, (2) partially directing, (3) passive supervisor, (4) partial initiator, (5) directed assisting and (6) non-involvement [33]. Especially when parents have a key role in the child’s diabetes care, parents are the guarantors of transfer. Parents encourage and prepare children to assume (more) responsibility for diabetes care and determine when children are “being given responsibility”. Parents are both responsible for facilitating the developmental task of independence as well as the child’s short-term and long-term health and well-being [17]. This means that parents need to navigate a delicate balance throughout the transfer of diabetes care responsibilities between on the one hand supporting the child’s autonomy and at the same time considering the child’s capability to assume responsibility.

Family Perspective

In the study focused on children with cystic fibrosis and their parents, parent and child roles appeared to move independently [33]. This means that parents and children may be in similar or different stages with respect to how ready they are to change the balance of responsibilities [43]. Child and parental perceptions of responsibility are ideally reciprocally discussed and synchronised, as dissimilar views about the balance of responsibility in parent–child dyads may result in:

- Parent–child conflicts, e.g., when children take over tasks when parents are not ready [21,44];
- Child emotional problems and suboptimal quality of care, for example when parents decrease their responsibility when children are not ready to assume more responsibility [21,27,44];
- Stagnations in the child’s development of independence, for example when children are ready to assume more responsibility but parents are not ready because of their own fear and sense of responsibility in preventing acute and long-term complications [44].

As children need the opportunity to learn [45], temporary reductions in the quality of diabetes care are normal and expected in the transfer process. However, these reductions may become problematic when parent and child roles remain diverged over longer periods of time.

Given that parents and children may have dissimilar views about how to divide tasks, the actual division of diabetes care responsibilities might not reflect the desired division by the parent and/or child. Therefore, the term “readiness” is often used to indicate how ready parents and children are to change the balance of responsibility [43]. In previous studies, the five stages of the transtheoretical model were used to describe and measure children’s and parents’ readiness to redistribute responsibilities, ranging from no interest in changing behaviours to actual behavioural change [43,46]. From a family perspective, the child’s responsibility ideally increases from, if applicable, solely parental responsibility to a responsibility that is shared between the parent(s) and child before it shifts to the child’s own responsibility [8,20].

### 3.2. Correlates of the Division and Transfer

Across families, variation is observed with regard to how responsibilities are divided between parents and children of the same age [47,48]. In some families, school-aged children have the responsibility for day-to-day tasks, whereas in other families parents are mainly responsible [17]. Several factors might explain this variation. Following the general framework of several leading developmental theories [49,50], these correlates and characteristics can be categorised into individual child, individual parent and context characteristics (Table 2). This premise that individual differences in functioning are multiply determined has already been established for other processes related to the transfer of diabetes responsibilities, for example:

- Childhood adaptation to diabetes. The “Childhood adaptation model to chronic illness” suggests that individual and family characteristics (age, sex, socioeconomic status, race/ethnicity, pubertal development, family environment, diabetes duration, treatment modality), psychosocial responses (stress, emotional/behavioural/eating problems), and individual/family responses (self-management, coping, self-efficacy, social competence, family functioning) are all relevant in this process [51].
- Transition to young adulthood with diabetes. This process is influenced by personal characteristics (e.g., depressive symptoms and impulse control but also diabetes-specific characteristics such as self-efficacy, fear of hypoglycaemia, perceptions about responsibility), environmental characteristics (e.g., parent–youth relationship, significant persons’ involvement), and transitional events (e.g., school changes, leaving parents’ home) [16].
- Resilience. The “diabetes resilience model” tries to understand why some youths who face the challenges of diabetes struggle, while others do well [52]. Among other things, it posits that individual, family, and social/context characteristics may function as risk and protective factors in explaining health outcomes. Furthermore, it states that both diabetes-specific and non-diabetes-specific (e.g., social and academic milestones) aspects of the lives of youths with type 1 diabetes may be of importance.

#### 3.2.1. Child Characteristics

Prior quantitative and qualitative studies have identified several individual child characteristics that are related to how diabetes care responsibilities are divided between parents and children [4,10,11,27,29,31,40,44,45,48,53,54,55,56,57,58,59,60,61,62,63,64,65,66,67,68,69,70,71,72,73,74,75,76,77,78,79,80,81,82,83,84,85,86,87,88,89,90,91,92,93,94,95]. These characteristics can be classified into the broader categories: sociodemographics, clinical characteristics, attachment, cognitive, emotional and social development, general autonomy, developmental disorders, diabetes related skills and knowledge, emotional well-being, willingness, diabetes self-care behaviours, diabetes perception and coping, diabetes self-efficacy, and temperament/behavioural problems.

Complementary to these findings, several theories were identified describing child characteristics that could be of importance for the division and transfer. The broader child developmental literature offers a range of models for conceptualising the physical, cognitive, social and emotional developments that are needed to assume diabetes care responsibilities [8,9]. Some of these theories state that children develop through subsequent developmental stages, such as Havighursts’s “Developmental tasks theory” [2], Piaget’s “Stages of cognitive development theory” [96], and Erikson’s “Theory of psychosocial development” [97]. Within diabetes care, these stages have been used to describe at what age children are expected to develop diabetes care related skills [9] and which challenges may interfere with the transfer of diabetes care responsibilities [8]. Children who have gone through a developmental stage or have completed developmental tasks successfully may adopt diabetes care responsibilities more easily [98].

Ryan and Deci’s “Self-determination theory” [13] has also been applied to the context of paediatric type 1 diabetes. It posits that effective diabetes self-care is stimulated by perceived competence (the judgement of one’s abilities to reach certain goals) and autonomous motivation (choosing a behaviour out of choice, satisfaction or pleasure, without outside pressures) [99].

The “developmental model of parent–child coordination for self-regulation” has been developed using paediatric type 1 diabetes management as an example [36]. This theory posits that parental behaviours, including behavioural involvement, and child regulatory skills important for diabetes care such as impulse control and emotional regulation dynamically influence each other over time to facilitate or hamper diabetes management. Periods of synchrony may alternate with periods of asynchrony, in which declining parental involvement may not always be in tune with adolescents’ competencies. In these cases, problematic blood glucose events like severe hypoglycaemia and prolonged hyperglycaemia often get the coordination between parental involvement and adolescent skill development back on track [36]. Additionally, it is stated that the child’s attachment to the parent is of importance for the coordination between parental involvement and child’s regulatory skills in diabetes care [36].

In “Bowlby’s attachment theory”, attachment is defined as the “lasting psychological connectedness between human beings” [100]. Initially, three types of attachment were defined: secure attachment, avoidant attachment and anxious attachment [101]. Later a fourth type, disorganised attachment, was added [102]. Children can experience the management of diabetes as complex and frustrating, which can be stressful and activate attachment behaviours [103]. As secure attachment contributes to the development of internal representations of self-worth and self-confidence, this type of attachment may stimulate a higher engagement with self-care as well as support seeking [103]. Insecure attachment may lead children to neglect themselves and their diabetes, which can hamper the transfer of diabetes care responsibilities [103]. Moreover, secure attachments with parents facilitate later relations with others, including the shift in interdependence with parents to friends and partners [36]. From a parental perspective, diabetes health risks may increase perceptions of a child’s vulnerability and activate worries about the child’s self-care abilities [104]. Especially in combination with any risky behaviours of the child, parents may experience separation anxiety and be triggered into overprotective attachment behaviours, potentially undermining their children’s autonomy [105]. However, when parents believe that their children are securely attached, it is easier for them to find a balance between parental involvement and child leadership [103].

#### 3.2.2. Parent Characteristics

Based on themes mentioned in previous qualitative studies and significant associations in quantitative studies, parent characteristics can be classified into the following categories [10,27,28,43,56,57,58,60,64,67,70,73,88,90,91,92,94,106,107,108,109,110]: sociodemographics, personality, negative emotions, parenting values/cognitions, diabetes self-efficacy and parenting behaviour.

As to existing theoretical models, Belsky’s “Process model of parenting” posited that next to characteristics of the child and context, the developmental history of the parent and parental personality play an important role in parenting [49]. In turn, parenting is proposed to be an important determinant of child development, together with the child’s characteristics. Learning theories also describe parenting behaviour as a key aspect of fostering the child’s development. In Vygotsky’s “sociocultural theory of cognitive development” it is stated that social learning (i.e., learning through interactions with more skilled others) is an important process through which children develop [111]. In this respect, parents may facilitate the learning process of their child by helping them with a task they can almost perform independently (“the zone of proximal development”) [112]. An important premise of Bandura’s “social cognitive theory” is that children may develop new skills through observation (“modelling” by others or following verbal instructions) and practicing [113]. If children learn how to cope with diabetes-related issues, they may develop a greater sense of diabetes-specific self-efficacy, i.e., their perceived confidence in their ability to perform diabetes care tasks [114,115].

As stated by the “self-determination theory”, active support from significant others, including parents, is needed for optimal motivation [99]. To this end, parental autonomy support acknowledges adolescents’ perspectives, provides meaningful information, and minimises pressure [99,116].

Moreover, parental monitoring is described as an important phase for parents in the transfer of diabetes care responsibilities in the “developmental model of parent–child coordination for self-regulation” [36]. Parental monitoring is focused on parental guidance of the child in performing diabetes tasks [117]. Monitoring involves regular contact, knowledge and supervision of the child’s diabetes care behaviours. This can be direct, in which the parent supervises a task the child is performing, or indirect, in which the parent asks the child about certain tasks he or she carried out [116]. Parental monitoring often occurs when responsibilities shift towards the child and the role of the parents changes from direct input to background evaluation and support [116]. As monitoring is solely a parent behaviour within the process of transfer (i.e., these behaviours are not transferred to children), this construct is not considered as part of the transfer itself.

Next to monitoring, it is also stated that the quality of the involvement is an important aspect within this process [36]. The quality of the involvement refers to how parents are involved in diabetes care and how this involvement is perceived by the child. The quality of involvement can be rated from high to low according to how helpful specific parental behaviours are in terms of following treatment recommendations, glycaemic outcomes, quality of life, and parent–child interactions such as collaborative partnership and family conflict [116]. High quality parental involvement focuses on the establishment of a collaborative partnership between child and parent through factors such as communication, warmth, sensitivity, emotional support, and independence encouragement [116]. An authoritative style of parenting combines high levels of warmth with moderate levels of parental control, allowing autonomy to learn while providing structure and support [118]. Authoritative parenting has been associated with a higher rate of following treatment recommendations, more optimal diabetes outcomes, and higher quality of life, and may also facilitate the child’s assumption of diabetes care tasks and the child’s acceptance of higher levels of parental involvement and monitoring [118,119]. Parental behaviours that are linked to a lower rate of self-care behaviours, suboptimal glycaemic outcomes, lower health-related quality of life and higher family conflict can be classified as lower quality parental involvement [116]. These parental behaviours are perceived as intrusive, overprotective, controlling, critical and harsh (e.g., lecturing, nagging, blaming, scolding, asking too many questions, restrictive strict rules/limits or giving orders), and provide low emotional support [116].

On the intersection between parent and child factors, factors relating to communication and (dis)agreement have been reported in quantitative and qualitative studies to be significantly related to the division and transfer [10,31,44,75,88,109,120]. The importance of (dis)agreement is further illustrated by two theories from the general child development literature dealing with the parent–child relationship. The “separation–individuation theory” posits that in order to develop and achieve a stable sense of self-identity, adolescents need to be able to become more independent from parent(s) and develop more autonomy [15]. The “autonomy–relatedness theory” additionally states that connection with parents remains important in this process [121,122]. Both perspectives argue that conflicts within the child–parent relationship may stimulate adolescent autonomy, provided that the parent–child relationship is realigned towards more appropriate expectations [123].

#### 3.2.3. Context Characteristics

In previous observational studies, a few context characteristics were identified that were associated with how diabetes care tasks were divided between parents and children [10,44,57,93,94,109]. These context characteristics can be categorised into those related to the family, school, work, health care team and peer group/social network.

These findings are in line with Bronfenbrenner’s “ecological systems theory” [50]. This theory states that children are embedded in several broader ecosystems. In previous models based on this theory within paediatric diabetes care focusing on diabetes treatment behaviours and the transition to adult care, these sources of support and stress included the broader family organisation (e.g., siblings, extended family), school, parental employment, peer group, health care system (including provider support and readiness, resources), and the larger economic, political and cultural spheres [124,125,126,127]. Context support might influence personal preferences with respect to responsibility division and might align parental and child readiness within the transfer process. For example, when diabetes teams regularly discuss the transfer, differences in the actual division and child readiness might be earlier identified and aligned. On the other hand, context demands may force families to take the next step in the transfer process, e.g., children might need to learn to assume responsibility for certain tasks because school personnel are no longer available to assist them at school [40,93].

#### 3.2.4. Interrelations Between Child, Parent and Context Characteristics

To some extent, children, parents and their surroundings (i.e., context) can be seen as single entities in the division and transfer of diabetes care responsibilities, as they can have a direct effect on child and parental readiness and the coordination between the two. For example, parental readiness might increase when children learn new skills. However, the interplay between characteristics of different domains can be observed on many levels. As a result, associations between a certain factor and the division/transfer could be the result of a shift in the balance of responsibility through or together with another characteristic. For example, the use of a sensor might help parents to increase autonomy supporting parenting, which might promote the child’s skills and knowledge to perform diabetes care tasks, which in turn could result in increased parental readiness and a shift from parental responsibility to shared responsibility.

**Table 2:** Overview of examples of child, parent and context characteristics that are likely to be associated with the division and transfer of diabetes care responsibilities, with supporting theories.

Category	Examples from Quantitative Studies	Examples from Qualitative Studies	Examples from Reviews Targeted to Children with Chronic Conditions in General	Supporting Theories
*Child characteristics*				
Sociodemographics	Age [4,27,29,30,31,56,57,58,59,60,61,62,63,64,66,67,68,69,70,71,72,73,76,128], sex [57]	Age [40,45,53,94], sex [55]	Age [10], sex [10], ethnicity [10]	
Clinical characteristics	Physical maturation [48,65,74,75,76], diabetes duration [27,64,68,76] age at diabetes diagnosis [69,74,77], pump use [37,75,78,79], years of pump use [69,80], age at pump start [80], HbA_1c_ [30,37,57,70,81,82,85,86], blood glucose variability [29], risk for glycaemic excursions [29], complexity of diabetes [64]	Age at onset [40], diabetes duration [94], sensor use [40,94], infuse/injection site [40,93], complications [40], predictability of diabetes [40], deterioration in health [40], blood glucose levels [40,94], perceived controllability/severity of hyper- and hypoglycaemia [40], hypoglycaemia awareness [40]	Disease duration and age at onset [10], co-morbid conditions [10], health status [10,11]	Developmental model of parent–child coordination for self-regulation [36]
Attachment				Developmental model of parent–child coordination for self-regulation [36], attachment theory [100]
Cognitive, social and emotional development	Conceptual ability [59], verbal working memory [59], memory [59], numeracy [87], social competence [37], executive dysfunction [95]	Memory/attention [40,94], affinity with technology [40], calculating [40,93], cognitive and emotional readiness [54]	Developmental stage [10], maturity [10], executive functioning [44]	Developmental tasks theory [2], stages of cognitive development theory [96], theory of psychosocial development [97]
General autonomy	General behavioural autonomy [72,83], functional autonomy [28], self-reliance [65]	Independence [40]	Personal autonomy [44]	
Developmental disorders		Autism [40]		
Diabetes related skills and knowledge	Diabetes problem solving [59], diabetes knowledge [59,66,89]	Mastery [40]	Disease knowledge [44]	
Emotional well-being	Depression [32,37], anger [37], diabetes related quality of life [77,84]	Fear and resistance [40], shame [40]	Fear of not “fitting-in” [10]	
Willingness	Perceived advantages and disadvantages of responsibility assumption [90]	Motivation [40,45]	Motivation [10,44]	Self-determination theory [13]
Diabetes self-care behaviours, diabetes perception and coping	Diabetes self-care behaviours [29,30,56,59,60,64,70,129], diabetes ownership [88], diabetes perception [84], diet avoidance [66]			
Diabetes self-efficacy	Diabetes self-efficacy [28,37,48,59,64,76,91]	Diabetes self-efficacy [40,92]	Diabetes self-efficacy [44]	Self-determination theory [13]
Temperament/behavioural problems	Behavioural problems [68]	Stubborn [40], assertive [40], worry [40], “bottle up” [40]		
*Parent characteristics*				
Sociodemographics	Maternal educational level [109]			
Developmental history				Process model of parenting [49]
Personality		Difficulty in ‘letting go’ [40,92]		Process model of parenting [49]
Negative emotions	Parenting stress [110], trait anxiety [58], depression [70]	Fear [40,94]	Fears of potential complications [10]	
Parenting values, cognitions and goals	Perceived disadvantages of adolescents’ assumption of diabetes management [90], independence priority [27], parenting goals [67], diabetes ownership [88], parental readiness to change the balance of responsibility [43,91]	Parenting values [40,94]	Attitudes [10]	
Diabetes self-efficacy	Diabetes self-efficacy [109]			
Parenting behaviour	Parental persuasive strategies [106], paternal autonomy support [28], parenting style [60], parental over-involvement diabetes [57], diabetes-specific autonomy support [107], family support for diabetes [73], supportive parental behaviour [64], non-supportive parental behaviour [64], frequency of parental help [108], parental involvement [56]	Parenting behaviours with the goal of a) promoting the child to assume (more) responsibility, b) handle child resistance if parents need to perform diabetes care tasks, c) relinquishing parental control, d) shape the environment if children are not yet capable to assume responsibility and parents are not present, e) optimise the transfer of diabetes care responsibility [40,92,94]	Communication style [10]	Developmental model of parent–child coordination for self-regulation [36], self-determination theory [13], process model of parenting [49], sociocultural theory of cognitive development [111], social cognitive theory [113]
*Parent–child interaction*				
Communication and (dis)agreement	Diabetes related conflict [75,120], conflict about responsibility division [109], problem solving [31], congruent perceptions of diabetes ownership [88]		Family conflict [44], communication [10]	Separation–individuation theory [15], autonomy–relatedness theory [121,122]
Connectedness				Autonomy–relatedness theory [121,122]
*Context characteristics*				
Family	Household composition [57], family history of diabetes [109]	Presence of parents [40], structure [40]	Household [10], prior experiences with older siblings [10]	Ecological systems theory [50]
School		Availability of instrumental support [40,94], anticipation of situations where children spend more time without parents [40,93]	Structure within school environment [10]	Ecological systems theory [50]
Work	Maternal employment status [57]			Process model of parenting [49], ecological systems theory [50]
Healthcare team	Diabetes centre [57]	Support healthcare team [40]	Support health care team [10]	Ecological systems theory [50]
Peer group/social network		Contact with other families [40], availability of instrumental support [40,54]		Process model of parenting [49], ecological systems theory [50]
Culture, economy, politics			Financial situation [44], social economic status [10]	Ecological systems theory [50]

### 3.3. How to Evaluate the Division of Diabetes Care Responsibilities

From conversations with parents about the transfer of diabetes care responsibilities, it becomes clear that the “most optimal” division or timing of responsibility transfer is difficult to define [40]. In line with the previously defined goal throughout the transfer process (i.e., “providing appropriate preparation for independence in adult life while ensuring the child’s safety and well-being”), a variety of concrete outcomes can be proposed for evaluating “success” (an overview can be found in Table 3). These outcomes stem from outcome measures used in previous models within paediatric diabetes care [16,51,52,126], as well as parental evaluation criteria of the division and transfer of responsibilities [40].

Several key points related to this selection of outcomes to evaluate the division and transfer stand out:

- The evaluation of the division may take place at the level of the individual child or within the parent–child interaction (i.e., conflicts, connectedness). Although parents also reported that the division and transfer could positively and negatively impact parent domains (e.g., energy level, level of parental monitoring and attendance with social activities, mood) and other family domains (e.g., attention for other children in the household), these consequences were considered to be part of parenting and were not mentioned as parental evaluation criteria of the division [40];
- The evaluation of the division may refer to different child domains: biomedical (i.e., HbA_1c_, blood glucose values, acute complications, diabetes-related hospital admissions, weight), behavioural (i.e., following or showing commitment towards treatment recommendations), emotional (i.e., emotional distress, [health-related] quality of life, perceived security/loneliness), and developmental (i.e., extent to which the child has a “normal” childhood/is not affected in his/her “normal” life by diabetes, independence level) [16,40,51,52,126];
- Outcomes may be diabetes-specific (e.g., HbA_1c_) or general (e.g., emotional distress);
- An apparently objective outcome may be subjective within itself (e.g., should HbA_1c_ values that do not meet the recommended target but do not further deteriorate during adolescence be considered as optimal outcomes?) [126];
- Outcomes may conflict amongst themselves, e.g., what is optimal for diabetes care may negatively affect the child enjoying a “normal” childhood [67];
- Children, parents and health care providers may have different views about the goals of diabetes care [130];
- The goal of clinical diabetes care is achieving and maintaining optimal, near normal blood glucose levels while taking quality of life into account [19,131];
- Satisfaction with the current division (e.g., too much or too little responsibility, appraisal of parental involvement) may be used to weigh and transcend outcomes from different domains [57,89,132,133,134].

### 3.4. Overreaching Conceptual Model

**Figure 1:**
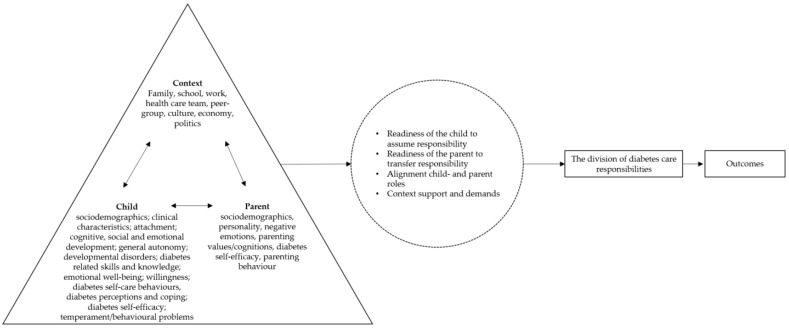
Proposed model to conceptualise the division of diabetes care responsibilities. In order to address the complexity of this process, bidirectional arrows were added between child, parent and context characteristics.

From the above literature review, the following premises can be distilled to inform an overarching conceptual model for understanding the division (at one point in time) and transfer (process over time) of paediatric diabetes care responsibilities:

- The division of diabetes care responsibilities (and most likely also the transfer) between parents and children with type 1 diabetes is multiply determined, with the child, parent, and context factors all playing a role (Table 2);
- These child, parent and context factors may relate to the division as single entities or in interplay with each other (Section 3.2.4);
- These factors likely relate to the division by affecting (a) the readiness of the child to assume responsibility (Section 3.1.2; e.g., support from the health care team can increase the child’s readiness to assume responsibility and result in more child responsibility), (b) the readiness of the parent to transfer responsibility (Section 3.1.2; e.g., high HbA1c values can decrease parents’ readiness to transfer responsibility and result in more parent responsibility), (c) the alignment of the child’s and the parent’s roles (Section 3.1.2; e.g., in a situation where the child is ready to take over a certain tasks but the parents are not ready to relinquish this task, the use of a sensor might enable parents to take a step back while they are not yet ready to “let go”, resulting in more child responsibility), and (d) pressures and support from the environment (Section 3.1; e.g., when the parent starts a new job where he/she can no longer be at home during the child’s lunch break and no one else is available to assist with the child’s diabetes self-care, the child needs to assume more responsibility although neither the parent nor the child is ready);
- The division of care responsibilities can be related to a myriad of outcomes (Section 3.3);

Child, parent, and context factors could be associated with outcomes through the division of care responsibilities (i.e., mediation). Figure 1 displays the model for the division of diabetes care responsibilities.

## 4. Discussion

In this narrative review, we have presented a conceptual model for understanding the division and transfer of diabetes care responsibilities between parents and children with type 1 diabetes. This model aids in the broader use and integration of the scattered knowledge in this field. Obviously, reality is more complex than any model can capture, and even the simplified version presented here is unlikely to be captured in a single study. However, similar to other theoretical models in this field, it is intended to guide researchers and diabetes care professionals in their thinking about these constructs [51].

The structure of the proposed conceptual model needs to be formally examined in further observational studies. In operationalising its subcomponents, researchers may draw on the suggestions made with respect to factors and outcomes and select those best fitting their specific research questions and the sample at hand. As illustrated, “responsibility” is a complex concept, defying easy and straightforward definition. Researchers are advised to consider what it is that they want to study (e.g., having responsibility, taking responsibility, shared responsibility), as the questions or approaches needed to elicit this information from families may differ. It might also be helpful to gain a better understanding of what our existing quantitative tools actually measure by asking children and parents what comes to their mind when completing these questionnaires. By (re)formulating questions as clearly as possible, measurement error due to different understandings of terms may be limited.

In the model itself, we have tried to do justice to the complexity of responsibility division and transfer, for example by drawing double-headed arrows between factors. However, in order to conclude with a workable model, we have had to exclude some potential premises. For example, we have framed the overall structure as a mediation model [27,51,57,70,108], while there are also indications that the division may act as a moderator [52,135]. Due to the scarcity and mixed findings of longitudinal studies [32,37,48,70,77,84,129], we have mainly focused on responsibility division (e.g., the division at a certain point in time) in the model. As stated before, responsibility transfer is a dynamic process over time in which feedback loops, non-linear associations, and temporary steps back are possible [40]. Mixed-method longitudinal research closely following a family’s decision processes over time may shed more light on the temporal aspects of responsibility transfer.

The translation of the conceptual model to clinical practice requires some further remarks. The highly individual character of the division and transfer of responsibility makes it difficult to apply a group model to specific families. However, the model might serve as a “roadmap” for discussing and tailoring these themes in regular care consultations. This may also shed more light on the interplay between factors. It is important to include both children and parent(s) in these conversations [136,137]. Parents have described their struggles with these topics and expressed a need for more support from diabetes care teams [40,138,139,140]. By having a joint conversation, discrepancies in readiness and roles can be identified and addressed [21]. The diabetes care team may have a facilitating role in this respect [134], while keeping in mind that families may have their own criteria to evaluate the “best” way of dividing responsibilities. Furthermore, interference by the diabetes care team is not in all cases appreciated by children and adolescents [10].

Having the conversation and improving the understanding of each other’s perspectives may by itself trigger a realignment of roles [21]. When barriers prove more complex and there is a need to intervene more structurally, several options are at hand to support families within the transfer of diabetes care responsibilities. Interventions focused on parent–child teamwork and communication [141], monitoring and follow-up by health care teams of glucose values [142], and self-care and parenting skills [143] have shown to affect certain aspects of the balance of diabetes care responsibilities. Multisystemic therapy (i.e., an intensive family-centred, community-based treatment originally designed for use with adolescents presenting with serious antisocial behaviour focused on youth following treatment demands) has previously been shown to reduce parental overestimation [144]. Moreover, by fostering facilitating factors such as child self-efficacy and motivation and tackling barriers including anxiety, both child and parental readiness may be increased. Apart from psychological approaches, technological devices aiding in diabetes care may be useful, such as starting with a sensor to monitor glucose and hybrid closed-loop systems [40]. Parents themselves may also play a key role in the child’s development towards autonomy, for example when child wants more responsibility but does not yet have the required skills, by shaping the environment to enable the child to assume responsibility or providing emotional support to deal with situations children struggle with. The process of finding new ways of supporting their developing child may be difficult for parents, as increases in child responsibility often are followed by (temporary) setbacks in glycaemic outcomes. Support programmes stimulating high quality involvement and autonomy supporting parenting behaviour may empower parents to be more effective coaches of their child in diabetes care. Peer support may be especially valuable in this respect [40].

## 5. Conclusions

The division of responsibilities is a multifaceted process that appears to be affected by a complex interplay of child, parent and context characteristics. These factors seem to change the division of diabetes care responsibilities by affecting (1) child/parental readiness to assume responsibility, (2) the alignment between child and parental readiness and (3) context support and demands. The “success” of the division and transfer of diabetes care responsibilities can be defined by biomedical, emotional, behavioural and parent–child interaction outcomes. The conceptual model presented in this narrative review provides a good stimulus for putting these constructs on the research and clinical agenda more structurally.

## Figures and Tables

**Table 1:** Definitions of concepts relevant in the division and transfer of diabetes care responsibilities.

Concept		Definition
Distribution/division		Refers to a static state—one point in time
Diabetes care responsibility		Duty or obligation of dealing with diabetes care tasks (“having/bearing responsibility”) in a way that conforms to norms (“assuming/taking responsibility”)
Division of diabetes care responsibilities within families		Who—parent or child—has the duty or obligation of dealing with a diabetes care task (“having/bearing responsibility”) in a way that conforms to norms (“assuming/taking responsibility”)
Child’s goal of the transfer of diabetes care responsibilities	Independent administration	Initiating and implementing health care tasks generally out of sight or supervision from a parent [11].
	Autonomy and independence (separation—independence)	Taking responsibility without relying on parents [12,13,14].
	Autonomy (self-determination)	Self-government based on personal interest, values and goals [12,15].
	Ownership/ primary diabetes care responsibility	Acting responsible while feeling it as one’s own obligation to do so [16,17].
	Shift in interdependence	Instead of parents, close friends and romantic partners provide support to assume responsibility for diabetes care [18].
Parental goal of the transfer of diabetes care responsibilities		An adult-to-adult relation between parents and children with regard to the child’s health; parents can still provide emotional and instrumental support on request, but they are no longer responsible for the quality and outcomes of diabetes care (“letting go”)
Transfer of diabetes care responsibilities		The transfer of diabetes care responsibilities is a process combining granting of more independence by parents (lowering parental role in diabetes care) and assuming of responsibilities by children (increasing child role in diabetes care).
Parental goal throughout the transfer process		Providing appropriate preparation for independence in adult life while ensuring the child’s safety and well-being.

**Table 3:** Possible outcome measures to evaluate the division and transfer of diabetes care responsibilities.

Outcome Level	Domain	Outcomes Within Models Within Paediatric Diabetes Care	Parental Evaluation Criteria of the Division and Transfer of Responsibilities
Child	Biomedical	HbA_1c_ [16,51,52,126], acute complications [126], hospital admissions [52]	(Changes in) HbA_1c_ [40], frequency of hypo- and hyperglycaemia [40], weight [40]
	Emotional	Emotional distress [126], (health-related) quality of life [16,51,52,126]	Security/loneliness [40]
	Behavioural	Following of treatment recommendations [16,52,126]	Commitment towards following treatment recommendations [40]
	Developmental		Extent to which the child can have a “normal” childhood/is affected by diabetes [40], independence level [40]
Parent–child interaction			Conflicts [40], connectedness [40]
